# Effects of platelet rich plasma (PRP) on human gingival fibroblast, osteoblast and periodontal ligament cell behaviour

**DOI:** 10.1186/s12903-017-0381-6

**Published:** 2017-06-02

**Authors:** Eizaburo Kobayashi, Masako Fujioka-Kobayashi, Anton Sculean, Vivianne Chappuis, Daniel Buser, Benoit Schaller, Forenc Dőri, Richard J. Miron

**Affiliations:** 1 Department of Cranio-Maxillofacial Surgery, University Hospital, University of Bern, Bern, Switzerland; 2 Department of Oral and Maxillofacial Surgery, School of Life Dentistry at Niigata, The Nippon Dental University, Niigata, Japan; 3 Department of Oral Surgery, Clinical Dentistry, Institute of Biomedical Sciences, Tokushima University Graduate School, Tokushima, Japan; 4 Department of Periodontology, School of Dental Medicine, University of Bern, Bern, Switzerland; 5 Department of Oral Surgery and Stomatology, School of Dental Medicine, University of Bern, Bern, Switzerland; 6 Department of Periodontology, Semmelweis University, Budapest, Hungary; 7 Department of Periodontology, College of Dental Medicine, Nova Southeastern University, Fort Lauderdale, FL USA; 8 Cell Therapy Institute, Center for Collaborative Research, Nova Southeastern University, Fort Lauderdale, FL USA

**Keywords:** Platelet rich plasma, Platelet concentrates, Growth factor release, Periodontal regeneration

## Abstract

**Background:**

The use of platelet rich plasma (PRP, GLO) has been used as an adjunct to various regenerative dental procedures. The aim of the present study was to characterize the influence of PRP on human gingival fibroblasts, periodontal ligament (PDL) cells and osteoblast cell behavior in vitro.

**Methods:**

Human gingival fibroblasts, PDL cells and osteoblasts were cultured with conditioned media from PRP and investigated for cell migration, proliferation and collagen1 (COL1) immunostaining. Furthermore, gingival fibroblasts were tested for genes encoding TGF-β, PDGF and COL1a whereas PDL cells and osteoblasts were additionally tested for alkaline phosphatase (ALP) activity, alizarin red staining and mRNA levels of osteoblast differentiation markers including Runx2, COL1a2, ALP and osteocalcin (OCN).

**Results:**

It was first found that PRP significantly increased cell migration of all cells up to 4 fold. Furthermore, PRP increased cell proliferation at 3 and 5 days of gingival fibroblasts, and at 3 days for PDL cells, whereas no effect was observed on osteoblasts. Gingival fibroblasts cultured with PRP increased TGF-β, PDGF-B and COL1 mRNA levels at 7 days and further increased over 3-fold COL1 staining at 14 days. PDL cells cultured with PRP increased Runx2 mRNA levels but significantly down-regulated OCN mRNA levels at 3 days. No differences in COL1 staining or ALP staining were observed in PDL cells. Furthermore, PRP decreased mineralization of PDL cells at 14 days post seeding as assessed by alizarin red staining. In osteoblasts, PRP increased COL1 staining at 14 days, increased COL1 and ALP at 3 days, as well as increased ALP staining at 14 days. No significant differences were observed for alizarin red staining of osteoblasts following culture with PRP.

**Conclusions:**

The results demonstrate that PRP promoted gingival fibroblast migration, proliferation and mRNA expression of pro-wound healing molecules. While PRP induced PDL cells and osteoblast migration and proliferation, it tended to have little to no effect on osteoblast differentiation. Therefore, while the effects seem to favor soft tissue regeneration, the additional effects of PRP on hard tissue formation of PDL cells and osteoblasts could not be fully confirmed in the present in vitro culture system.

## Background

Various regenerative modalities in modern dental medicine have been investigated in recent years with the aim of either speeding hard or soft tissue regeneration or optimising the regenerative outcomes [1–4]. One of these modalities frequently promoted has been the utilization of growth factors including platelet derived growth factor (PDGF) and bone morphogenetic proteins (BMPs) [4, 5]. While the use of such growth factors has been shown to speed the quality of either hard or soft tissue formation specifically when combined with various biomaterials including barrier membranes and bone grafting materials, some disadvantages such as high costs, high supra-physiological doses of growth factors, as well as unwanted side effects associated with recombinant therapies have also been reported [6–8].

Interestingly, the use of platelet rich plasma (PRP) contains plasma with up to a 5-fold increase in platelet concentrations [9], has been shown to improve growth factor concentrations from whole blood by centrifugation to reach supra-physiological doses [10, 11]. Reported advantages include having higher biocompatibility as well as relatively low costs associated with their treatment [12–17]. Initial experiments revealed that PRP contained high levels of PDGF compared to whole blood capable of modulating tissue wound healing [9, 17–21]. Other investigators have hypothesized that bone healing could be improved due to the angiogenetic, proliferative and/or differentiating effects of PRP on various cell types [19, 22–24]. While initial experiments hypothesized over such advantages, data from the literature have shown mixed results following the use of PRP as a regenerative material for bone and periodontal regeneration [17, 25–39].

Since PRP has now been utilized in the field of periodontology for over a decade with various results, the aim of the present study was to perform one of the largest known in vitro studies on the topic by utilizing 3 different cell-types involved with periodontal regeneration including human gingival fibroblasts, osteoblasts and periodontal ligament (PDL) cells. Currently, there remains controversial results for studies that have attempted to determine the bone-healing and soft tissue-healing potential of PRP. Therefore, this in vitro study was utilized to better characterize the regenerative potential of conditioned media from a new formulation of PRP (GLO PRP) on each cell type with direct comparison over their regenerative potential being possible within the same study.

## Methods

### Platelet concentrates

Blood samples were harvested with the informed consent of 6 volunteer donors (from Bern Switzerland) and further processed into PRP. No IRB was required for the present study as blood was used in a non-identifiable manner and the IRB waived its requirement (Bern, Switzerland). The PRP was prepared utilizing the GLO PRP kit. Briefly, 1 ml of sodium citrate anticoagulant was added into the GLO PRP tube and 9 ml of blood was then taken from the donor. Thereafter the red blood cell collector was attached firmly on the tip of the GLO PRP tube and the plunger removed by turning it counter clockwise and the blood + anticoagulant was shaken gently. The first centrifuge was then performed at 1200 g for 5 min and thereafter the remaining red blood cells were flushed out from the tube to separate them. The remaining tube was re-centrifuged for a second time at 1200 g for 10 min. Thereafter, platelet poor plasma (PPP) and PRP were separated using a 5 ml syringe with needle to collect the PPP. The remaining PRP was further utilized for experimental seeding as later indicated. Blood was collected from members of our laboratory (30 to 60 years of age).

Thereafter PRP was transferred to 6 well culture dishes with 5 mL of cell culture media and processed for further investigation as previously described [40–42]. PRP was incubated for 3 days in a spinning chamber at 37 °C and conditioned media (CM) was collected and utilized for future experiments as a 20% total volume dose.

### Protein quantification with ELISA

ELISA was utilized to quantify the total amount of growth factors released from PRP at 15 min, 60 min, 8 h, 1 day, 3 days and 10 days. All PRP samples were harvested and placed in a shaking incubator at 37 °C where growth factors were gradually released over time and collected from standard tissue culture media. At each time point, media was collected and replaced with a fresh 5 mL of media and frozen for later processing. Standing ELISA was utilized to quantify proteins according to the manufacturer’s protocols as previously described [43]. Absorbance was measured at 450 and 570 nm on an ELx808 Absorbance Reader (BIO-TEK, Winooski, VT) and samples were quantified in triplicate with 3 independent experiments performed.

### Cell isolation and culture

Primary human gingival tissues were obtained from three healthy donors under-going third molar extraction as previous described [44]. Similarly, primary osteoblasts were also cultured from bone tissue during removal of impacted 3^rd^ molars as previously described [45, 46]. Primary human PDL cells were harvested from the middle third portion of healthy extracted teeth (for normal standard care) with no signs of periodontal disease extracted for orthodontic reasons as previously described [45, 46]. Cells from each tissue type (1. gingival fibroblasts, 2. osteoblasts and 3. PDL cells) were collected from 3 separate donors and pooled with similar cell-types. No IRB was required for the present study as cells were collected in a non-identifiable manner and the IRB waived its requirement (Bern, Switzerland). An ethical approval with informed written consent was obtained from all patients. All cells were detached from tissue culture plastic using 0.25% EDTA-Trypsin (Gibco, Life technologies, Carlsbad, CA) prior to reaching confluency. Cells were cultured in a standard humidified atmosphere at 37 °C in of DMEM (Gibco), 10% fetal bovine serum (FBS; Gibco) with 1% antibiotics (Gibco). Cells (passage 4–6) were seeded with 20% conditioned media from PRP for experimental purposes as previously described [47]. 10,000 cells were utilized for cell proliferation experiments and 50,000 cells were utilized for real-time PCR, immunofluorescent staining, ALP assay and alizarin red experiments in 24 well culture dishes.

### Cell viability

Primary human gingival fibroblasts, PDL cells and osteoblasts were seeded at a density of 10’000 cells per 24-well plate on control tissue culture plastic, with 20% conditioned media from PRP and 10% FBS. At 24 h post cell seeding, a live-dead staining assay was used to assess cell viability (Enzo Life Sciences AG; Lausen, Switzerland) as previously described [48]. Thereafter, the percentage of live versus dead cells was utilized to quantify cell viability.

### Cell migration assay

A Boyden chamber was utilized to investigate migration of cells using 24-well plates and polyethylene terephthalate filters with a pore size of 8 μm (ThinCertTM, Greiner Bio-One GmhH, Frickenhausen, Germany). PRP conditioned media (20%) in DMEM containing 10% FBS was placed into the lower wells as previously described [40, 49]. After starving the cells in DMEM containing 0.5% FBS for 12 h, 10,000 cells were seeded in the upper compartment and after 24 h, the number of migrated cells on the lower side of the filter were counted under a microscope as previously described [40, 49].

### Proliferation assay

Ten thousand primary human gingival fibroblasts, PDL cells and osteoblasts were seeded in 24-well culture plates with 20% conditioned media from PRP. An MTS assay was utilized to quantify cells (Promega, Madison, WI) at 1, 3 and 5 days as previously described [46, 50]. At desired time points, cells were washed with phosphate buffered solution (PBS) and quantified using a ELx808 Absorbance Reader.

### Real-time PCR analysis

Total RNA was harvested at 3 and 7 days post stimulating for gingival fibroblasts to investigate mRNA levels of TGF-β, PDGF-A, PDGF-B and collagen1a2 (COL1a2). For PDL cells and osteoblasts, osteoblast differentiation markers including Runx2, COL1a2, alkaline phosphatase (ALP) and osteocalcin (OCN) mRNA levels were investigated at 3 and 14 days post-seeding. Table 1 reports the primer and probe sequences for genes. RNA isolation (High Pure RNA Isolation Kit, Roche, Switzerland) and real-time RT-PCR (Roche Master mix on an Applied Biosystems 7500 Real-Time PCR machine) was then performed. A Nanodrop 2000c (Thermo, Wilmington, DE) was utilized to calculate total RNA levels. The ∆∆Ct method was utilized to calculate gene expression levels normalized to the expression of GAPDH.

**Table 1:** List of primer sequences for real-time PCR

Gene	Primer Sequence
hTGF-β F	actactacgccaaggaggtcac
hTGF-β R	tgcttgaacttgtcatagatttcg
hPDGF-A F	cacacctcctcgctgtagtattta
hPDGF-A R	gttatcggtgtaaatgtcatccaa
hPDGF-B F	tcccgaggagctttatgaga
hPDGF-B R	actgcacgttgcggttgt
hCOL1a2 F	cccagccaagaactggtatagg
hCOL1a2 R	ggctgccagcattgatagtttc
hRunx2 F	tcttagaacaaattctgcccttt
hRunx2 R	tgctttggtcttgaaatcaca
hALP F	gacctcctcggaagacactc
hALP R	tgaagggcttcttgtctgtg
hOCN F	agcaaaggtgcagcctttgt
hOCN R	gcgcctgggtctcttcact
hGAPDH F	agccacatcgctcagacac
hGAPDH R	gcccaatacgaccaaatcc

### ALP activity assay

PDL cells and osteoblasts were stimulated with 20% conditioned media from PRP with and without osteogenic differentiation medium (ODM), which consisted of DMEM supplemented with 10% FBS, 1% antibiotics, 50 μg/ml ascorbic acid (Sigma, St. Louis, MO) and 10 mM β-glycerophosphate (Sigma) to induce osteoblast differentiation as previously described [51]. The ALP assay was performed using the Leukocyte alkaline phosphatase kit (procedure No. 86, Sigma) as previously described [45, 52–55].

### Mineralization assay

Alizarin red staining was used to determine osteoblast mineralization. After 14 days, cells were stained followed by 0.2% alizarin red solution staining (pH 6.4) as previously described [45, 50, 51, 56, 57].

### Collagen immunofluorescent staining

Human gingival fibroblasts, PDL cell and osteoblasts were plated at a density of 50,000 cells per structure in a 24-well plate containing 20% conditioned media from PRP. At 14 days post seeding, cells were fixed and stained with collagen type I antibodies (sc-28657, Santa Cruz, California, USA) diluted 1:100 in PBS containing 1% BSA as previously described.

### Statistical analysis

Three independent experiments performed in triplicate were utilized for all conditions. Means and standard errors (SE) were calculated and data were analyzed for statistical significance using standard t-test (2 groups), one way analysis of variance (ANOVA) with Tukey test by GraphPad Prism 6.0 software.

## Results

### Release of growth factors from PRP

**Fig. 1:**
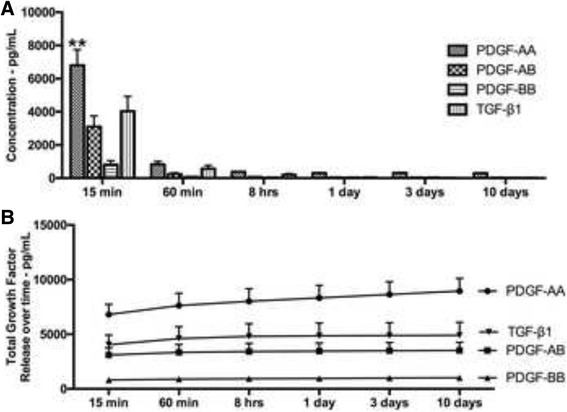
Growth factor release from PRP over time. **a** growth factor release of PDGF-AA, PDGF-AB, PDGF-BB and TGF-β1 at various time points. **b** Total growth factor release over a 10-day period

In a first series of experiments, the total amount of growth factors released from PRP including PDGF-AA, PDGF-AB, PDGF-BB, TGF-β1, VEGF, IGF and EGF was investigated at various time points including 15 min, 60 min, 8 h, 1 day, 3 days, and 10 days. It was found that of all the growth factors, PDGF-AA released the highest total amount of growth factor followed by TGF-β1 (Fig. 1). Interestingly, most of the growth factor release occurred at early time points (15 and 60 min), whereas little release of growth factor was seen at later time points up to a 10 day period. Figure 1b demonstrates the total growth factor release accumulated in the culture media over a 10 day period. The release of VEGF, IGF and EGF followed similar trends whereby growth factor release occurred at early time points (15 and 60 min) and was maintained up to a 10 day period (data not shown). The total growth factor release from each donor is summarized in Table 2 along with the minimum, maximum and average values.

**Table 2:** Total growth factors released over 10 days from PRP. Data represents averages (pg/ml) with ranges (minimum to maximum values)

	PRP – minimum	PRP – average	PRP - maximum
PDGF-AA	5968	8954	15787
PDGF-AB	1644	3522	8179
PDGF-BB	415	1020	2761
TGF-β1	366	4909	12414
VEGF	187	331	486
EGF	77	182	344
IGF	511	987	1728

### Cell viability of gingival fibroblasts, PDL cells and osteoblasts in response to PRP

**Fig. 2:**
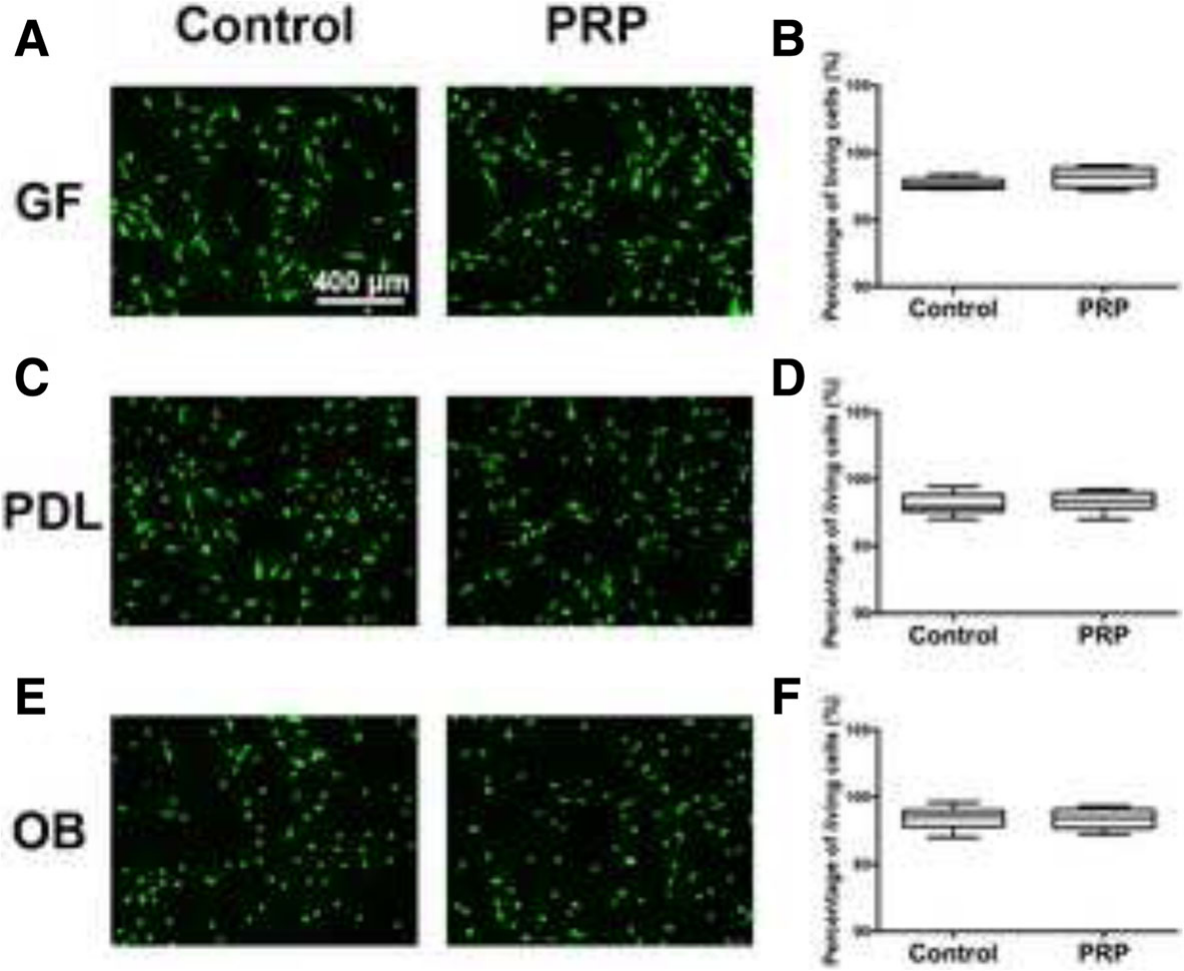
Live/Dead assay of PRP. Live/Dead assay at 24 h of primary human gingival fibroblasts (GF) (**a**, **b**), PDL cells (PDL) (**c**, **d**) and osteoblasts (OB) (**e**, **f**) in response to cell culture with PRP. No significant changes in cell viability were observed for all platelet concentrates

**Fig. 3:**
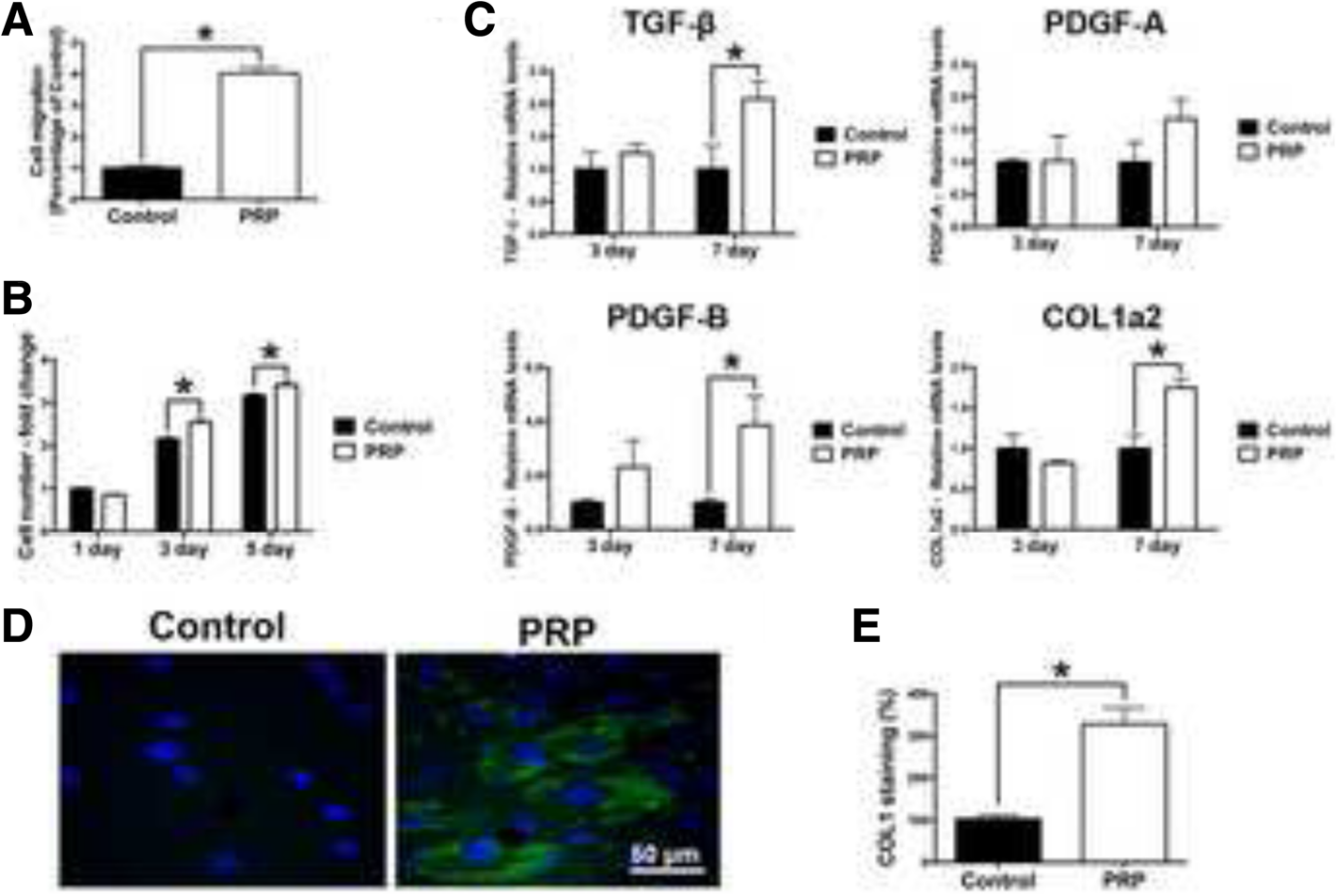
Effect of PRP on human gingival fibroblasts. Human primary gingival fibroblast cultured with PRP on (**a**) cell migration at 24 h, (**b**) proliferation at 1, 3 and 5 days, (**c**) real-time PCR at 3 and 7 days for mRNA levels of TGF-β, PDGF-A, PDGF-B and COL1a2, as well as (**d**, **e**) immunofluorescent collagen1 (COL1) staining at 14 days. (Data presents means and standard error bars; * denotes significant difference between groups, *p* < 0.05)

**Fig. 4:**
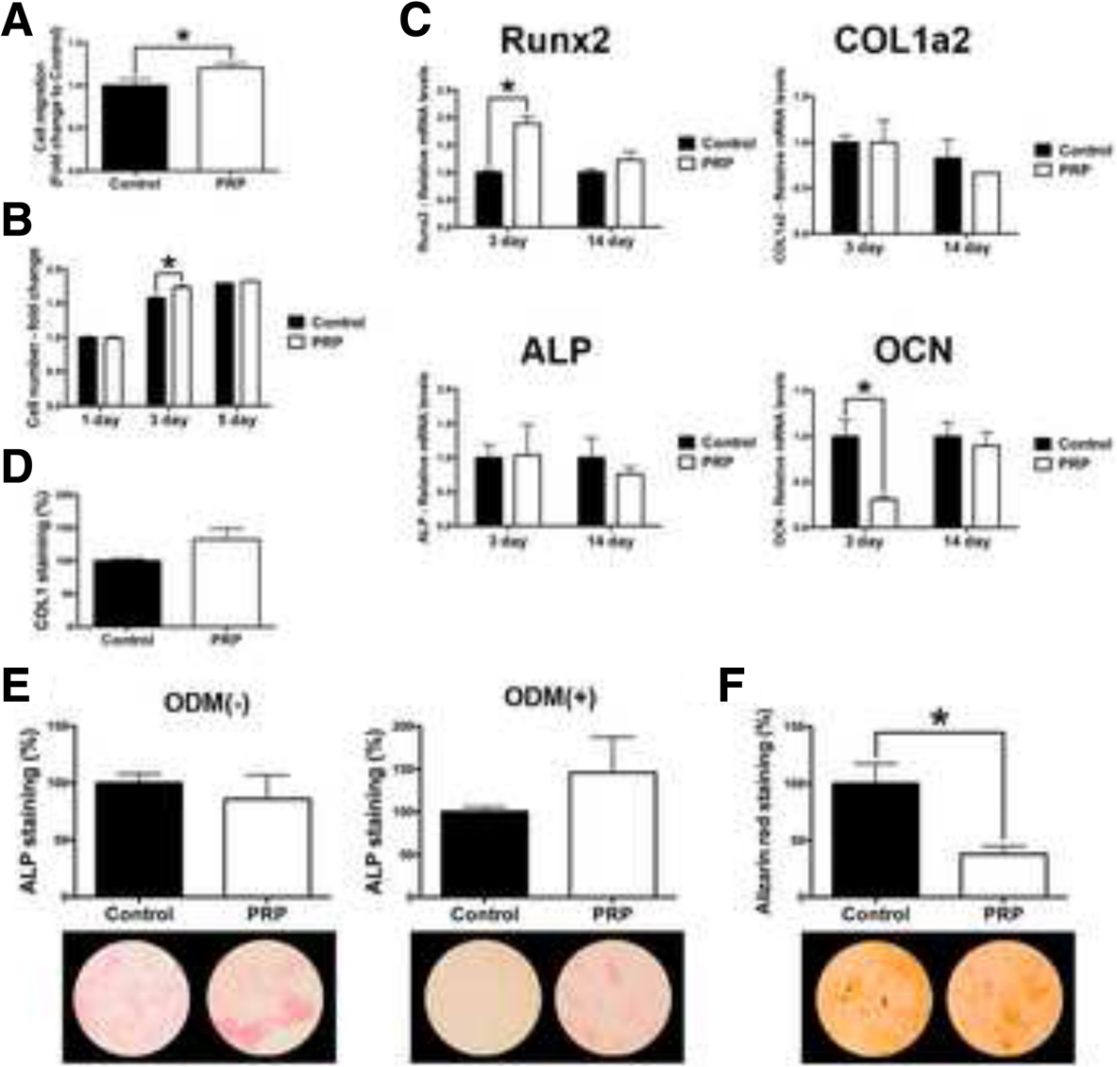
Effect of PRP on human periodontal ligament cells. Human PDL cells cultured with PRP on (**a**) cell migration at 24 h, (**b**) cell proliferation at 1, 3 and 5 days, (**c**) real-time PCR at 3 and 14 days for mRNA levels of osteoblast differentiation markers including Runx2, COL1a2, ALP and OCN, (**d**) immunofluorescent COL1 staining at 14 days, (**e**, **f**) ALP staining both with and without osteoblast differentiation media (ODM) at 14 days, as well as (**f**) alizarin red staining denoting mineralization at 14 days post seeding. (Data presents means and standard error bars; * denotes significant difference between groups, *p* < 0.05)

**Fig. 5:**
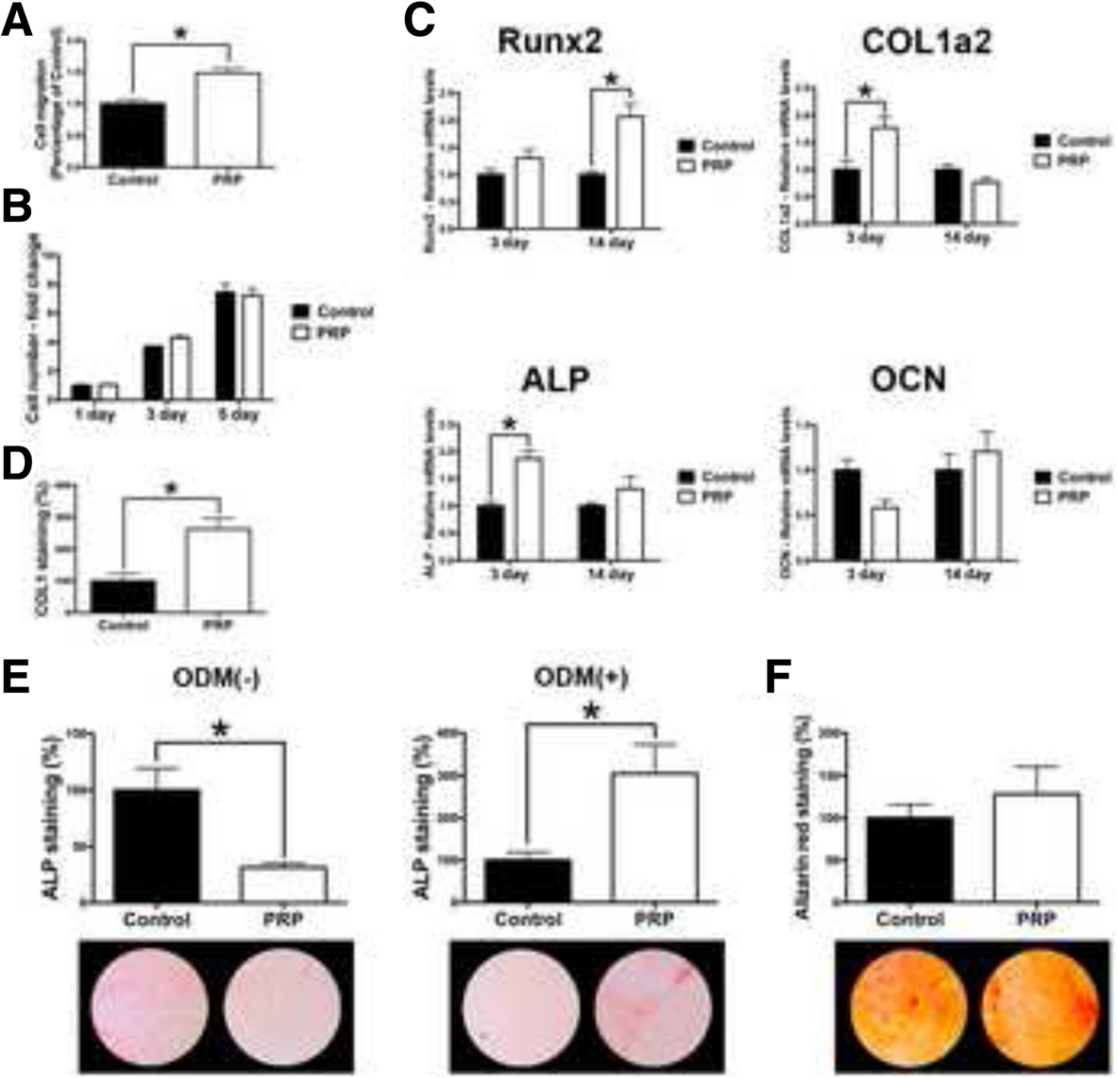
Effect of PRP on human osteoblasts. Human osteoblasts cultured with PRP on (**a**) cell migration at 24 h, (**b**) cell proliferation at 1, 3 and 5 days, (**c**) real-time PCR at 3 and 14 days for mRNA levels of osteoblast differentiation markers including Runx2, COL1a2, ALP and OCN, (**d**) immunofluorescent COL1 staining at 14 days, (**e**, **f**) ALP staining both with and without osteoblast differentiation media (ODM) at 14 days, as well as (**f**) alizarin red staining denoting mineralization at 14 days post seeding. (Data presents means and standard error bars; * denotes significant difference between groups, *p* < 0.05)

In a first set of experiments, we sought to characterize the influence of conditioned media from PRP on cell viability of 3 cell types including gingival fibroblasts, PDL cells and osteoblasts (Fig. 2). It was found that all cells displayed no significant changes in cell viability following 24 h cell culture with and without PRP. Therefore, it was initially confirmed that PRP is fully biocompatible under the present in vitro cell culture model and future experiments were designed thereafter to investigate its effect on cell behaviour (Figs. 3, 4 and 5).

### Influence of PRP on human gingival fibroblasts

Following cell survival investigation, conditioned media from PRP was then investigated on gingival fibroblast cell behaviour (Fig. 3). It was first observed that PRP induced a 4-fold significant increase in cell migration of gingival fibroblasts at 24 h (Fig. 3a). Furthermore, PRP also significantly increased cell proliferation of gingival fibroblasts at 3 and 5 days post seeding (Fig. 3b). Real time PCR demonstrated that PRP significantly increased regenerative mRNA levels of growth factors including TGF-β, PDGF-B and COL1a2 at 7 days post seeding (Fig. 3c). Immunofluorescent staining of COL1 at 14 days further demonstrated a 300% increase in COL1 staining when compared to gingival fibroblasts cultured on control tissue culture plastic without PRP (Fig. 3d, e).

### Influence of PRP on human PDL cells

Thereafter, conditioned media from PRP was investigated on PDL cell behaviour (Fig. 4). It was found that PRP significantly increased cell migration at 24 h (Fig. 4a) and cell proliferation at 3 days (Fig. 4b) however the effects were much less pronounced when compared to gingival fibroblasts. Real time PCR for osteoblast differentiation markers including genes encoding Runx2, COL1a2, ALP and OCN did not seem to show any trend towards improving their differentiation towards osteoblasts/cementoblasts by demonstrating no changes in COL1a2 and ALP mRNA levels at both 3 and 14 days post seeding (Fig. 4c). Interestingly, PRP significantly increased mRNA levels of Runx2 at 3 days, however not at 14 days, whereas mRNA levels of OCN were significantly down-regulated at 3 days with no differences found at 14 days (Fig. 4c).

Thereafter, COL1 immunofluorescent staining as well as ALP staining showed no preference for PRP when compared to control tissue culture plastic (Fig. 4d, e). PRP also failed to promote the mineralization potential of PDL cells at 14 days by demonstrating significantly lower levels of alizarin red staining at 14 days post seeding (Fig. 4f).

### Influence of PRP on human osteoblasts

Lastly, the effect of conditioned media from PRP was investigated on human osteoblasts (Fig. 5). It was first observed that PRP significantly increased osteoblast migration at 24 h (Fig. 5a). No effect on osteoblast proliferation was seen at either 1, 3 or 5 days post seeding (Fig. 5b). Real-time PCR demonstrated that PRP significantly increased COL1a2 and ALP mRNA levels at 3 days, as well as Runx2 mRNA levels at 14 days post seeding (Fig. 5c). No significant difference in OCN mRNA levels was observed at either 3 or 14 days post-seeding following osteoblast culture with PRP (Fig. 5c).

Thereafter, it was found that PRP significantly promoted a 2.5 fold increase in COL1 immunofluorescent staining at 14 days post seeding (Fig. 5d). Interestingly, ALP staining without ODM found that PRP significantly decreased ALP staining whereas with ODM significantly promoted ALP staining at 14 days (Fig. 5e). No change in alizarin red staining was observed following osteoblast culture with PRP (Fig. 5f).

## Discussion

The aim of the present study was to investigate the in vitro regenerative potential of conditioned media from PRP on 3 different cell-types involved in periodontal regeneration including gingival fibroblasts, PDL cells and osteoblasts. To date, no study has performed such a widespread in vitro analysis on the performance of PRP (from GLO systems) on these cell-types. Furthermore, new spin cycle protocols and various formulation of platelet concentrates are routinely brought to market and it is therefore of clinical interest and significance to investigate their individual performances [15, 47, 58, 59].

Recently, we investigated the release of various growth factors from 3 different platelet concentrates [58]. It was first found that PRP, in comparison to PRF, released higher amounts of growth factors including PDGF, TGF-β and VEGF at early time points (15 and 60 min) and therefore favoured the early release of growth factors for regenerative procedures [58]. In the present study, we also found that this different formulation of PRP, spun with pre-existing columns to separate platelet concentrates, also formulated a PRP with a release profile of growth factors similar to previous formulations of PRP. Interestingly however, a slight increase in total growth factor, most likely resulting from the present system utilized was noticed.

The concentrated growth factors released from PRP was then investigated on the migration and proliferation of various cell-types from the oral cavity (Figs. 2, 3 and 4). Interestingly, it was found that gingival fibroblasts demonstrated the ability to further promote cell migration and proliferation in response to PRP when compared to PDL cells and osteoblasts. Furthermore, COL1 staining was also more significantly upregulated in gingival fibroblasts when compared to PDL cells and osteoblasts. Interestingly, it was observed that PRP had no effect on the ALP activity of PDL cells and actually significantly down-regulated in vitro mineralization potential of PDL cells by demonstrating lower levels of alizarin red staining (Fig. 4) whereas its effect on osteoblasts demonstrated little to no changes in mineralization potential (Fig. 5). It may therefore be concluded that while PRP showed little to no potential to induce osteoblast differentiation (new bone formation), its effects seemed to favour gingival fibroblast regeneration (soft tissue wound healing). Future animal models are however necessary to further confirm these in vitro findings.

Over the years, numerous investigations have been performed to determine the regenerative potential of PRP for both soft and hard tissue regeneration [60–62]. For instance, Graziani et al. previously investigated the biological rationale of PRP by evaluating its effect at different concentrations on fibroblasts and osteoblasts activity in vitro [62]. It was found that PRP preparations exerted a dose-specific effect on oral fibroblasts and osteoblasts. Interestingly, increased concentrations resulted in a reduction in proliferation and a suboptimal effect on osteoblast function primarily [62]. Griffin et al. showed in a systematic review that although early clinical results suggest that the use of PRP is safe and feasible, however presents with no clinical benefit in either acute or delayed fracture healing was observed and therefore its use in bone regeneration was not undetermined [61]. While other models have also shown favorable results on new bone formation with platelet concentrates [63–65], the results from our study showed that PRP had little influence on osteoblast differentiation (Fig. 5). Therefore and in combination with some of the previous published studies, it may be suggested that PRP induced a strong potential for soft-tissue regeneration by demonstrating marked increases in gingival fibroblast cell migration, proliferation, release of growth factor and collagen synthesis (Fig. 3) yet has no obvious potential to induce or enhance new bone formation. Although several advantages existed when PRP was combined with PDL cells and osteoblasts including increased cell migration, proliferation or collagen synthesis, these marked increases were less pronounced and did not seem to contribute to their differentiation or mineralization potential at least in vitro.

## Conclusions

The results from the present study demonstrated that PRP is able to release supra-physiological doses of platelet-derived growth factors including PDGF-AA, PDGF-AB, PDGF-BB, TGF-β1, VEGF, EGF and IGF at varying concentrations for up to 10 days. While PRP was extremely biocompatible with all cell-types, it can be hypothesized that PRP is able to induce greater soft tissue regeneration by significantly increasing the stimulation of gingival fibroblast behaviour when compared to PDL cells or osteoblasts. Little effects, however, were observed for PRP on osteoblasts differentiation or mineralization potential of PDL cells and osteoblasts in vitro. Future animal testing is thus required to further evaluate the regenerative potential of PRP in both soft and hard tissue formation.

## Abbreviations

ALP: Alkaline phosphatase
BMP2: Bone morphogenetic protein 2
COL1: Collagen1
EGF: Epidermal growth factor
ELISA: Enzyme-Linked Immunosorbent Assay
IGF: Insulin growth factor
IRB: Internal Review Board
OCN: Osteocalcin
ODM: Osteogenic differentiation medium
PBS: Phosphate buffered solution
PDGF: Platelet-derived growth factor
PDL: Periodontal Ligament
PPP: Platelet poor plasma
PRP: Platelet rich Plasma
SE: Standard error
TGFβ1: Transforming growth factor Beta 1
TGFβ1: Transforming growth factor beta-1
VEGF: Vascular endothelial growth factor
