# Erratum to: Effects of platelet rich plasma (PRP) on human gingival fibroblast, osteoblast and periodontal ligament cell behaviour

**DOI:** 10.1186/s12903-017-0388-z

**Published:** 2017-06-13

**Authors:** Eizaburo Kobayashi, Masako Fujioka-Kobayashi, Anton Sculean, Vivianne Chappuis, Daniel Buser, Benoit Schaller, Ferenc Dőri, Richard J. Miron

**Affiliations:** 1 Department of Cranio-Maxillofacial Surgery, University Hospital, University of Bern, Bern, Switzerland; 2 Department of Oral and Maxillofacial Surgery, School of Life Dentistry at Niigata, The Nippon Dental University, Niigata, Japan; 3 Department of Oral Surgery, Clinical Dentistry, Institute of Biomedical Sciences, Tokushima University Graduate School, Tokushima, Japan; 4 Department of Periodontology, School of Dental Medicine, University of Bern, Bern, Switzerland; 5 Department of Oral Surgery and Stomatology, School of Dental Medicine, University of Bern, Bern, Switzerland; 6 Department of Periodontology, Semmelweis University, Budapest, Hungary; 7 Department of Periodontology, College of Dental Medicine, Nova Southeastern University, Fort Lauderdale, FL USA; 8 Cell Therapy Institute, Center for Collaborative Research, Nova Southeastern University, Fort Lauderdale, FL USA

## Erratum

“Upon publication of the original article [1], it was noticed that there was an error in the author name. The author’s name should be “ Ferenc” instead of “Forenc”.
